# The Trade-Off Between Format Familiarity and Word-Segmentation Facilitation in Chinese Reading

**DOI:** 10.3389/fpsyg.2021.602931

**Published:** 2021-01-28

**Authors:** Mingjing Chen, Yongsheng Wang, Bingjie Zhao, Xin Li, Xuejun Bai

**Affiliations:** Key Research Base of Humanities and Social Sciences, Institute of Psychology and Behavior, Tianjin Normal University, Tianjin, China

**Keywords:** trade-off, format familiarity, word segmentation, Chinese reading, eye movements

## Abstract

In alphabetic writing systems (such as English), the spaces between words mark the word boundaries, and the basic unit of reading is distinguished during visual-level processing. The visual-level information of word boundaries facilitates reading. Chinese is an ideographic language whose text contains no intrinsic inter-word spaces as the marker of word boundaries. Previous studies have shown that the basic processing unit of Chinese reading is also a word. However, findings remain inconsistent regarding whether inserting spaces between words in Chinese text promotes reading performance. Researchers have proposed that there may be a trade-off between format familiarity and the facilitation effect of inter-word spaces. In order to verify this, this study manipulated the format familiarity via reversing the Chinese reading direction from right to left to investigate this issue in Experiment 1 and Experiment 2. The purpose of Experiment 1 was to examine whether inter-word spaces facilitated Chinese reading in an unfamiliar format. Experiment 1 was conducted that 40 native Chinese undergraduates read Chinese sentences from right to left on four format conditions. The results showed faster reading speed and shorter total reading time for the inter-word spaced format. Based on this finding, Experiment 2 examined whether the facilitation effect of inter-word spaces would reduce or disappear after improving the format familiarity; this experiment was conducted that 40 native Chinese undergraduates who did not participate in Experiment 1 read Chinese sentences from right to left on four format conditions after ten-day reading training. There was no significant difference between the total reading time and reading speed in the inter-word spaced format and unspaced format, which suggests that the facilitation effect of inter-word spaces in Chinese reading changed smaller. The combined results of the two experiments suggest that there is indeed a trade-off between format familiarity and the facilitation of word segmentation, which supports the assumption of previous studies.

## Introduction

As the saying goes, “Read wide, and you will wisely write.” In other words, reading is the basic way for humans to acquire information, as well as an effective way to inherit human knowledge and civilization. Different civilizations have produced different languages with different characteristics. For example, Chinese is an ideographic language that differs from an alphabetic language like English, whose texts contain intrinsic inter-word spaces as word boundary markers. The basic independent unit of reading processing whether in Chinese or English is the word (Rayner, 1998, 2009; Rayner et al., 1998; Reichle et al., 1999; Inhoff et al., 2000; Yan et al., 2006; Li et al., 2009; Perea and Acha, 2009). The primary task for readers of Chinese is to segment words from Chinese texts which do not contain inter-word spaces as an indicator of word segmentation. The characteristic of no inter-word spaces has been the subject of many studies focusing on the mechanism of word segmentation in Chinese reading (Rayner, 1998; Bai et al., 2008; Bassetti, 2009; Li et al., 2009, 2014; Cui et al., 2014; Zang et al., 2016; Ma, 2017; Liu and Lu, 2018; Ma and Zhuang, 2018; Zhou et al., 2020). Moreover, the reading direction is one of the significant characteristics among languages. In some languages, such as Hebrew and Arabic, text by default is read from right to left (Deutsch and Rayner, 1999). The default direction of ancient Chinese texts was also from right to left and from top to bottom. However, the direction of modern Chinese texts has changed to a left to right direction, which is a familiar reading format for native Chinese readers. This poses the following questions: when reading texts with an unfamiliar format which differs from the default format (for example, Arabic readers whose default format is reading from right to left in arabic texts read Chinese texts from left to right in unfamiliar format), how do readers understand and process the information given in the unfamiliar format? Does reading performance change under unfamiliar format? Are there any differences between reading processing in the unfamiliar format and the familiar format? These questions need to be explored in psychology of reading.

It is a subject of debate whether inter-word spaces as indicators of word segmentation could promote Chinese reading. Many studies have supported inter-word spaces being explicit indicators that promote word recognition and reading processing (Yan et al., 2010; Li et al., 2011a; Zang et al., 2013; Bai et al., 2015). However, some researchers did not find evidence of inter-word spaces facilitating reading; a representative study is that by Bai et al. (2008). In this study, the segmentation was manipulated to present Chinese sentences in different formats (unspaced format, inter-word spaced format, inter-character spaced format, non-word spaced format) to native Chinese readers in natural reading. However, the results did not completely support the research hypothesis: there was no facilitation effect of inter-word spaces. To explain this finding, it was proposed that there may be a trade-off between format familiarity and word-segmentation facilitation in Chinese reading. This means that inter-word spaces do indeed facilitate Chinese reading; however, the format unfamiliarity of inserting spaces into texts offsets the facilitation of word segmentation. To verify this proposal, the following studies found that inter-word spaces facilitated Chinese reading for foreign students and children with dyslexia (Bai et al., 2009; Wang et al., 2010; Shen et al., 2012; Zang et al., 2013). What these participants had in common was a low level of proficiency in Chinese reading; in addition, they were unfamiliar with the format of unspaced Chinese texts, which was caused by their insufficient reading experience. Therefore, inter-word spaces facilitated reading in this unfamiliar format. However, the studies using Chinese native readers as participants did not find that inter-word spaces promoted the reading process in the familiar format (Shen et al., 2010; Yan et al., 2012). In summary, the format familiarity of Chinese text was the distinguishing factor between the studies finding that inter-word spaces facilitated reading and those that found no evidence of facilitation.

Based on previous findings, this study manipulated word segmentation and format familiarity to investigate whether there is a trade-off between format familiarity and word-segmentation facilitation in Chinese reading. In Experiment 1, the Chinese sentences were presented in four segmented formats (unspaced format, inter-character spaced format, inter-word spaced format, non-word spaced format). Chinese readers were required to read from right to left, which was an unfamiliar format for them. In Experiment 2, the reading training (reading the Chinese texts from right to left) was manipulated to improve participants’ familiarity with the format. Following this, the same Chinese sentences were presented in the same four segmented formats and the Chinese readers were required to read from right to left after having improved their familiarity with the format. Based on the assumption that there is a trade-off between format familiarity and word-segmentation facilitation in Chinese reading, if the results of Experiment 1 showed that inserting spaces between words did indeed facilitate the reading process, this would prove that inter-word spaces did facilitate reading in the unfamiliar format. On the other hand, if inserting spaces between words did not promote Chinese reading, it would imply that inter-word spaces did not facilitate reading in the unfamiliar format.

After obtaining the results in Experiment 1, the study turned to Experiment 2. The unfamiliar format was changed to a familiar format via reading training in Chinese reverse texts. If the facilitation of word segmentation reduced or disappeared under this condition, it would verify that the facilitation was offset or partly offset by the format familiarity. On the other hand, if nothing changed with the facilitation of word segmentation, then it would imply that the format familiarity did not affect inter-word spaces facilitating Chinese reading by giving word-segmentation indications.

## Experiment 1

The purpose of Experiment 1 was to determine whether inter-word spaces facilitate Chinese reading in an unfamiliar format without relevant reading experience.

### Method

#### Participants

The participants in Experiment 1 were 40 undergraduate students (mean ages 20.78 ± 1.21 years). There were 28 females and 12 males; their mother tongue was Chinese; they had normal or corrected vision; and they were right-handed dominant. Each participant signed a project agreement form before the experiment commenced. Based on the Declaration of Helsinki (BMJ 1991; 302: 1194), Tianjin Normal University’s Medical Ethical Committee approved the experiment.

#### Experimental Design and Materials

The experimental design was a single-factor (word segmentation) within-subjects design which contained four conditions: (1) Unspaced format: Chinese default format with no spaces in sentences; (2) Inter-character spaced format: spaces between adjacent characters in sentences; (3) Inter-word spaced format: spaces between words in sentences; (4) Non-word spaced format: spaces were randomly inserted between sentences to turn adjacent words into non-words. Examples of sentences in the four conditions are shown in Table 1.

**TABLE 1:** Example Chinese stimuli from the four conditions used in the experiment.

Word segmentation	Sentence example
Unspaced format	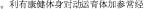
Inter-character spaced format	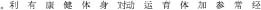
Inter-word spaced format	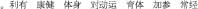
Non-word spaced format	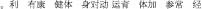

*The English translation for the sentence is “Regular participation in sports is good for health.” “
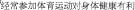
” is the Chinese sentence in the normal format.*

A total of 60 Chinese sentences were constructed, which all ranged between 15 and 17 characters in length (M = 15.8 characters, SD = 0.80 characters). The experimental sentences were rated on a seven-point scale for their naturalness by 30 participants who did not take part in the eye-tracking experiment. The mean naturalness score was 6.69 (where a score of 7 was very natural) and the consistency of the word segmentation in sentences was 91% by 12 participants who did not take part in the eye-tracking experiment. Experiment 1 constructed four files, each of which had 60 sentences. The 60 sentences were allocated to the four conditions, which were rotated in the form of a Latin square and contained 15 sentences. The blocked format was the presented format of sentences in each condition, which had a random order. Each file contained 12 practical sentences which was allocated to four conditions, each of which contained three sentences. The 12 practical sentences were presented to be read first in each file. Participants needed to answer yes/no after each of 22 sentences, after which followed a comprehension question where the number of yes and no responses was equal. Each participant read 72 sentences in total.

#### Apparatus

The experiment recorded right-eye movements using EyeLink 1000 (SR Research, Canada); the sampling rate was 1,000 Hz, while the accuracy rate was a 0.5° visual angle. We adjusted the resolution of the stimulus presented on a 19-inch Dell monitor to 1,024 × 768. In the experiment, participants maintained a distance of 70 cm from the screen. The characters had a size of 25 × 25 pixels and the visual angle was 0.80°, and they were presented in sentences in the Song font.

#### Procedure

The participants were told to read sentences from right to left in different conditions before the experiment. Participants needed to understand the meaning of sentences as quickly as possible and press the space bar to read the next sentence. In some sentences a comprehension question followed, which participants had to then answer as correctly as possible. Chin rests were used to ensure that participants’ heads remained in a resting position to compensate for head movement. A calibration was completed before the experiment to calculate the position of the fixed point. Participants started the test after successful calibration. If necessary, the eye location would be recalibrated during the experiment. The experiment lasted about 20 min. The participants’ responses to the comprehension questions achieved a correctness rate of 91.0%, which indicated that the sentences had been read and understood.

#### Data Preparation and Analysis

According to the following criteria (Rayner et al., 2006; Bai et al., 2008; Rayner, 2009; Liu et al., 2017; Wang et al., 2018), the analysis of fixation durations excluded data shorter than 80 ms and longer than 800 ms. The data were excluded if: (1) participants pressed the key incorrectly during the experiment, which resulted in an interruption; (2) data were lost due to accidental factors (such as head movement); (3) there were fewer than four gazes; (4) the data were outside three standard deviations. After excluding invalid data (2.8% of the total data), data analysis was conducted.

The experiment computed eye-movement measures of target words as follows: (1) Mean fixation duration (average fixation duration in all fixated points in the sentence); (2) Mean saccadic length (average length of all saccades from the current fixated point to the next one in the sentence); (3) Number of fixations (number of all fixations in the sentence); (4) Total fixation duration (sum of the fixation duration in all fixation points in the sentence); (5) Reading speed (average number of reading words per second in the sentences); (6) Forward saccadic length (saccadic length reading from right to left). The units of the time index (mean fixation duration; total fixation duration) were milliseconds; the units of the mean saccadic length and forward saccadic length were characters; while characters per second were the units of reading speed. SPSS 20 for Windows (SPSS Inc., Chicago, IL, United States) was used to process statistical data. A repeated-measures analysis of variance, including subject analysis (F_1_) and item analysis (F_2_), was conducted (Shen et al., 2010; Bai et al., 2012a, 2015; Zang et al., 2013).

### Results

The results of the global analysis are shown in Tables 2A, 2B, 3.

**TABLE 2A:** Global eye movement measure under four conditions.

	Unspaced format	Inter-character spaced format	Inter-word spaced format	Non-word spaced format
Mean fixation duration	262 (43)	236 (34)	241 (39)	242 (36)
Mean saccadic length	2.62 (1.03)	4.10 (1.38)	3.75 (1.32)	3.56 (1.25)
Number of fixations	14.4 (5.55)	15.68 (5.60)	14.11 (5.14)	14.86 (5.31)
Total fixation duration	4385 (1817)	4412 (1732)	3993 (1583)	4312 (1749)
Reading speed	4.31 (2.11)	4.17 (1.85)	4.60 (2.04)	4.33 (2.01)
Forward saccadic length	2.16 (1.42)	3.19 (1.98)	2.97 (1.85)	2.89 (1.85)

*The averages in the table and the standard deviations in parentheses. The unit of mean fixation time is milliseconds; the unit of mean saccade length is character; the unit of total time is milliseconds; the unit of reading speed is character per second; the unit of forward saccade length is character.*

**TABLE 2B:** The 95% confidence intervals of global eye movement measure under four conditions.

		Unspaced format		Inter-character spaced format		Inter-word spaced format		Non-word spaced format	
		Lower	Upper	Lower	Upper	Lower	Upper	Lower	Upper
Mean fixation duration	*F*_1_	252	270	229	242	232	248	234	249
	*F*_2_	259	266	234	239	238	244	239	245
Mean saccadic length	*F*_1_	2.38	2.89	3.84	4.63	3.46	4.14	3.30	3.97
	*F*_2_	2.54	2.71	4.00	4.25	3.64	3.88	3.47	3.67
Number of fixations	*F*_1_	13.01	15.57	14.24	16.79	12.87	14.97	13.54	15.82
	*F*_2_	13.67	15.17	14.75	16.56	13.36	14.69	14.23	15.50
Total fixation duration	*F*_1_	3894	4745	3939	4766	3583	4287	3583	4287
	*F*_2_	4110	4621	4112	4686	3751	4183	4091	4532
Reading speed	*F*_1_	3.84	4.91	3.77	4.68	4.17	5.13	3.88	4.87
	*F*_2_	4.07	4.61	3.92	4.49	4.36	4.90	4.09	4.62
Forward saccadic length	*F*_1_	2.05	2.57	3.06	3.92	2.83	3.51	2.75	3.44
	*F*_2_	2.13	2.24	3.16	3.35	2.93	3.10	2.86	3.03

*The averages in the table and the standard deviations in parentheses. The unit of mean fixation time is milliseconds; the unit of mean saccade length is character; the unit of total time is milliseconds; the unit of reading speed is character per second; the unit of forward saccade length is character. Lower means lower confidence limit, upper means upper confidence limit. F_1_ means the subject analysis, F_2_ means the item analysis.*

**TABLE 3:** F values, degrees of freedom, *p* values, η^2^ values, and *post hoc* comparisons for each index.

Index	*F*		*df*	*p*	η^2^	*Post hoc* comparisons, *p* < 0.05
Mean fixation duration	*F*_1_	61.82	3,117	<0.001	0.61	Condition 1 > Condition 2, Condition 1 > Condition 3;
	*F*_2_	59.46	3,177	<0.001	0.50	Condition 1 > Condition 4; Condition 4 > Condition 2
Mean saccadic length	*F*_1_	188.44	3,117	<0.001	0.77	Condition 2 > Condition 1, Condition 2 > Condition 3; Condition 2 > Condition 4;
	*F*_2_	149.32	3,177	<0.001	0.68	Condition 3 > Condition 1; Condition 3 > Condition 4; Condition 4 > Condition 1;
Number of fixations	*F*_1_	11.61	3,117	<0.001	0.23	Condition 2 > Condition 1, Condition 2 > Condition 3,
	*F*_2_	3.90	3,177	0.010	0.062	Condition 2 > Condition 4; Condition 4 > Condition 3;
Total fixation duration	*F*_1_	10.88	3,117	<0.001	0.22	Condition 3 < Condition 1, Condition 3 < Condition 2, Condition 3 < Condition 4;
	*F*_2_	2.82	3,177	0.04	0.05	
Reading speed	*F*_1_	10.84	3,117	<0.001	0.22	Condition 3 > Condition 1, Condition 3 > Condition 2, Condition 3 > Condition 4;
	*F*_2_	1.85	3,177	0.14		
Forward saccadic length	*F*_1_	128.84	3,117	<0.001	0.77	Condition 2 > Condition 1, Condition 2 > Condition 3,
	*F*_2_	127.65	3,177	<0.001	0.68	Condition 2 > Condition 4; Condition 3 > Condition 1;

*The unit of mean fixation time is milliseconds; the unit of mean saccade length is character; the unit of total time is milliseconds; the unit of reading speed is character per second; the unit of forward saccade length is character. F_1_ means the subject analysis, F_2_ means the item analysis. Condition 1 means the Unspaced format, Condition 2 means the inter-character spaced format, Condition 3 means the inter-word spaced format, Condition 4 means the non-word spaced format.*

The results showed a significant word-segmentation effect in the mean fixation duration: *F*_1_(3,117) = 61.82, *p* < 0.001, η^2^ = 0.61; *F*_2_(3,177) = 59.46, *p* < 0.001, η^2^ = 0.50. Furthermore, the results of the *post hoc* test showed no significant difference between the inter-word spaced condition and the non-word spaced condition, *p* > 0.05. The difference between inter-character spaced condition and inter-word spaced condition was also not significant, *p* > 0.05. There were significant differences between each of the other two conditions, *p*_*s*_ < 0.05. The mean fixation duration of the unspaced format was the longest, *p_*s*_* < 0.001. The mean fixation duration of the inter-character spaced format was the shortest, *p*_*s*_ < 0.05. This meant that participants took a shorter duration to process words and understand the Chinese sentences. Inserting spaces into characters in Chinese sentences could reduce the difficulty of Chinese reading.

In Chinese, the saccade length, which reflects the language information in the unit space, is generally only two to three Chinese characters (Inhoff and Liu, 1998; Yan and Bai, 2007; Yan et al., 2013). A longer saccade length indicates that the participants obtained relatively more information during the saccade fixation (Irwin, 1998; Yan and Bai, 2000; Yan et al., 2013). There was a significant word-segmentation effect in the mean saccadic length: *F*_1_(3,117) = 188.44, *p* < 0.001, η^2^ = 0.83; *F*_2_(3,177) = 149.32, *p* < 0.001, η^2^ = 0.72. The results of the *post hoc* test showed that there was a significant difference between each of the two conditions, *ps* < 0.01. The mean saccadic length in the inter-character spaced format was the longest, *ps* < 0.001. The mean saccadic length in the inter-word spaced format was longer than that in the unspaced format which was the shortest, *p* < 0.001. The main effect of word segmentation was significant in the forward saccadic length format, *F*_1_ (3,117) = 128.84, *p* < 0.001, η^2^ = 0.77; *F*_2_ (3,177) = 127.65, *p* < 0.001, η^2^ = 0.68. Furthermore, the results of the *post hoc* test showed that forward saccadic length in the inter-character spaced format was the longest, significantly longer than in the other conditions, *ps* < 0.001. Forward saccadic length in the inter-word spaced format was significantly longer than that of the unspaced format, *p* < 0.001. The forward saccadic length in the non-word spaced format was significantly longer than in the unspaced format, *p* < 0.05, which was marginally shorter than that in the inter-word spaced format, *p* = 0.068.

The number of fixations refers to the number of all fixation points, which could reflect the cognitive load of the reading material (Yan et al., 2013). The more difficult and complex the reading materials are, the higher the number of fixations (Henderson and Ferreira, 1990; Yan et al., 2013). The higher the reading level, the lower the number of fixations for the same reading material (Rayner et al., 2011). We found that there was a significant word-segmentation effect in the number of fixations: *F*_1_(3,117) = 11.61, *p* < 0.001, η^2^ = 0.23; *F*_2_(3,177) = 3.90, *p* = 0.010, η^2^ = 0.062. The results of the *post hoc* test showed that the number of fixations in the inter-character spaced format was the highest, *p_*s*_* < 0.05. The number of fixations in the inter-word spaced format was less than that of the non-word spaced format, *p* < 0.05. The difference between other conditions was not significant, *p*_*s*_ > 0.05. The number of fixations in the inter-word spaced condition was significantly less than that under non-word spaced condition, which may be due to non-word spaces interfering with word segmentation and increasing the difficulty of reading.

The total reading time is sensitive to slower and longer cognitive processing, which can reflect the processing difficulty of reading sentences (Rayner et al., 2011; Yan et al., 2013). In the results, the main effect of word segmentation was significant, *F*_1_(3,117) = 10.88, *p* < 0.001, η^2^ = 0.22; *F*_2_(3,177) = 2.82, *p* < 0.05, η^2^ = 0.05 The results of the *post hoc* test showed that the total fixation duration in the inter-word spaced format was the shortest of the four conditions, *p_*s*_* < 0.01. The difference between each of the other conditions was not significant, *p*_*s*_ > 0.05. This implies that inter-word spaces as word-segmentation clues could shorten the reading time and reduce the difficulty of Chinese texts. The reading speed represented the Chinese characters read per second. A faster reading speed means faster processing to understand the words and sentences and a lower difficulty with word recognition and reading comprehension. The results showed that the main effect of word segmentation on reading speed was significant, *F*_1_(3,117) = 10.84, *p* < 0.001, η^2^ = 0.22; *F*_2_(3,177) = 1.849, *p* = 0.140. Furthermore, the results of the *post hoc* test showed that the reading speed in the inter-word spaced format was significantly faster than in the other conditions, *ps* < 0.05. The difference between each other conditions was not significant, *p*_*s*_ > 0.05. The reading speed in the inter-character spaced format was slower than that in the unspaced format, but the difference between them was not significant, *p_*s*_* > 0.05. The reading speed on inter-character spaced format was slowest, which was not significant with that on the unspaced format and non-word spaced format, *p_*s*_* > 0.05. This showed that the inter-word spaces as word boundaries provided visual clues that facilitated Chinese reading and improved the reading speed, which is a finding consistent with previous results (Bai et al., 2009, 2015, 2014; Shen et al., 2010, 2012; Yan et al., 2010; Li and Pollatsek, 2011; Li et al., 2011b; Zang et al., 2013). In sum, the results in experiment 1 supported the research hypothesis.

### Discussion

The purpose of Experiment 1 was to determine whether inter-word spaces could facilitate Chinese reading in an unfamiliar format without relevant reading experience. We used four word-segmentation conditions to compare the eye movements of participants reading Chinese sentences from right to left in an unfamiliar format.

The previous results did not show the effect of inter-word spaces facilitating Chinese reading (Bai et al., 2008). The assumption proposed was that there was a trade-off between format unfamiliarity and facilitation effect of inter-word spaces in Chinese reading. The results showed that inserting spaces into words as indicators of word segmentation facilitated Chinese reading in the unfamiliar format, which supports the assumption. The logic behind this finding is that if there is a trade-off between format unfamiliarity and the facilitation of inter-word spaces, then the facilitation effect of inter-word spaces is offset by the format unfamiliarity of inserting spaces between words. However, the texts in the unspaced format had the format familiarity of the Chinese default format. There was no difference in the total reading time and reading speed between the inter-word spaced format and unspaced format. Therefore, if readers were both unfamiliar with the unspaced format and inter-word spaced format, the facilitation effect of inter-word spaces would not be offset by the unfamiliarity of the format. The results showing the total reading time was shorter and the reading speed was faster in the inter-word spaced format compared to the unspaced format indeed supported the assumption that there was a trade-off between format unfamiliarity and the facilitation effect of inter-word spaces in Chinese reading. In addition, the total reading time in the inter-word spaced format was shorter than that in the inter-character spaced format and non-word spaced format, which had the longest reading time. This may be due to the basic unit of information processing Chinese being a word rather than a character.

According to the results in Experiment 1, the readers’ familiarity with the format affected the facilitation effect of inter-word spaces; the inter-word spaces significantly facilitated Chinese reading where readers lacked reading experience of reverse text from right to left. We can therefore assume that with increased reading experience of Chinese reversed texts, the facilitation effect of inter-word spaces would gradually decrease or even disappear. This problem will be further verified in Experiment 2.

## Experiment 2

Experiment 1 showed that the inter-word spaces facilitated Chinese reading in an unfamiliar format in participants without relevant reading experience. Based on this finding, Experiment 2 focused on whether the facilitation effect of inter-word spaces would change with increased reading experience from right to left following reading training. Therefore, the combined results of Experiment 1 and 2 will demonstrate whether there is a trade-off between format unfamiliarity and the facilitation effect of inter-word spaces in Chinese reading.

### Method

#### Participants

The participants in Experiment 2 were 40 undergraduate students who did not participate in Experiment 1 (mean ages 20.50 ± 1.63 years). There were 31 females and 9 males; their mother tongue was Chinese; they had normal or corrected vision; and they were right-handed dominant. Each participant signed a project agreement form before the experiment commenced. Based on the Declaration of Helsinki (BMJ 1991; 302: 1194), Tianjin Normal University’s Medical Ethical Committee approved the experiment.

#### Experimental Design and Materials

The experimental design was a single-factor (word segmentation) within-subjects design which contained four condition, same as that in experiment 1. Examples of sentences in the four conditions are shown in Table 1. The materials used for the eye-movement tests were the same as in Experiment 1.

The materials in the reading training were 60 Chinese essays (average number of words M = 936) chosen from Chinese high-school textbooks, which were reversed from left–right format to right–left format through reversing software (see the Appendix for examples of reading materials).

#### Apparatus

The apparatus was same as that in Experiment 1.

#### Procedure

The procedure involved stages of reading training and eye movement. Firstly, participants entered the laboratory and were made familiar with the environment every day. Then the participants sat in their own seats where the experiment book was presented, which contained essays that the participants needed to read every day. The participants then read essays. Before the reading commenced, the researcher would give the following instruction: “Below you will read some articles. The sentences in the article will be presented from right to left. Please read carefully word by word and understand the article as much as possible. Seven reading comprehension questions will appear after each article. You are required to select the most appropriate answer based on the article and fill in the answer.” The participants began to read an article after understanding the instruction and then answered seven questions, before moving on to the next question.

The reading training lasted for 30 min every day for ten days. After 10 days of reading training, eye-movement testing began, followed the same procedure as Experiment 1. The participants’ responses to the comprehension questions in the eye-movement stage achieved a correctness rate 93.0%, which indicated that the sentences had been read and understood the sentences seriously.

#### Data Preparation and Analysis

According to the following criteria which were same as that in Experiment 1 (Rayner et al., 2006; Bai et al., 2008; Rayner, 2009; Li et al., 2017; Liang et al., 2017; Wang et al., 2018), analysis data was selected. After excluding invalid data (1.65% of the total data), data analysis was conducted.

The experiment computed eye-movement measures of target words which were same as that in the experiment 1. The same method in experiment 1 was used to process the statistical data. A repeated-measures analysis of variance, including subject analysis (F_1_) and item analysis (F_2_), was conducted (Shen et al., 2010; Bai et al., 2012a, b, 2015; Yu et al., 2018).

### Results

The results of the global analysis are shown in Tables 4A, 4B, 5.

**TABLE 4A:** Global eye movement measure under four conditions.

	Unspaced format	Inter-character spaced format	Inter-word spaced format	Non-word spaced format
Mean fixation duration	260 (37)	237 (34)	236 (37)	243 (37)
Mean saccadic length	2.07 (0.73)	3.45 (1.15)	2.80 (0.95)	2.73 (0.95)
Number of fixations	10.60 (3.52)	12.17 (3.96)	11.16 (3.66)	11.77 (4.07)
Total fixation duration	3116 (1087)	3313 (1103)	3043 (1034)	3266 (1122)
Reading speed	5.55 (1.98)	5.20 (1.78)	5.65 (1.87)	5.25 (1.88)
Forward saccadic length	2.21 (1.17)	3.34 (1.74)	2.98 (1.50)	2.88 (1.41)

*The averages in the table and the standard deviations in parentheses. The unit of mean fixation time is millisecond; the unit of mean saccadic length is character; the unit of total time is millisecond; the unit of reading speed is character per second; the unit of forward saccadic length is character.*

**TABLE 4B:** The 95% confidence intervals of global eye movement measure under four conditions.

		Unspaced format		Inter-character spaced format		Inter-word spaced format		Non-word spaced format	
		Lower	Upper	Lower	Upper	Lower	Upper	Lower	Upper
Mean fixation duration	*F*_1_	252	268	229	244	228	244	235	251
	*F*_2_	256	264	233	240	232	240	239	247
Mean saccadic length	*F*_1_	1.87	2.17	3.12	3.63	2.57	2,94	2.46	2.85
	*F*_2_	1.95	2.08	3.31	3.46	2.68	2.83	2.57	2.75
Number of fixations	*F*_1_	9.80	11.31	11.23	13.11	10.30	11.99	10.83	12.63
	*F*_2_	10.25	11.02	11.75	12.62	10.85	11.47	11.41	12.19
Total fixation duration	*F*_1_	2863	3376	3067	3623	2791	3338	3014	3543
	*F*_2_	3000	3263	3188	3452	2941	3143	3156	3391
Reading speed	*F*_1_	5.16	6.28	4.78	5.70	5.23	6.33	4.89	5.84
	*F*_2_	5.41	5.94	5.04	5.46	5.56	6.00	5.15	5.59
Forward saccadic length	*F*_1_	2.12	2.53	3.21	3.87	2.88	3.33	2.75	3.21
	*F*_2_	2.17	2.28	3.30	3.43	2.94	3.05	2.82	2.96

*The averages in the table and the standard deviations in parentheses. The unit of mean fixation time is milliseconds; the unit of mean saccade length is character; the unit of total time is milliseconds; the unit of reading speed is character per second; the unit of forward saccade length is character. Lower means lower confidence limit, upper means upper confidence limit. F_1_ means the subject analysis, F_2_ means the item analysis.*

**TABLE 5:** F values, degrees of freedom, *p* values, η^2^ values, and *post hoc* comparisons for each index.

Index	*F*		*df*	*p*	η^2^	*Post hoc* comparisons, *p* < 0.05
Mean fixation duration	*F*_1_	76.60	3,117	<0.001	0.66	Condition 1 > Condition 2; Condition 1 > Condition 3; Condition 1 > Condition 4;
	*F*_2_	25.70	3,177	<0.001	0.30	Condition 4 > Condition 2; Condition 4 > Condition 3;
Mean saccadic length	*F*_1_	209.98	3,117	<0.001	0.84	Condition 2 > Condition 1; Condition 2 > Condition 3; Condition 2 > Condition 4;
	*F*_2_	221.88	3,177	<0.001	0.79	Condition 3 > Condition 1; Condition 4 > Condition 1;
Number of fixations	*F*_1_	28.31	3,117	<0.001	0.42	Condition 2 > Condition 1; Condition 2 > Condition 3; Condition 4 > Condition 1;
	*F*_2_	21.96	3,177	<0.001	0.27	Condition 4 > Condition 3; Condition 3 > Condition 1;
Total fixation duration	*F*_1_	18.01	3,117	<0.001	0.42	Condition 3 < Condition 2; Condition 3 < Condition 4;
	*F*_2_	6.05	3,177	0.001	0.09	Condition 1 < Condition 2; Condition 1 < Condition 4;
Reading speed	*F*_1_	20.92	3,117	<0.001	0.35	Condition 3 > Condition 2; Condition 3 > Condition 4;
	*F*_2_	5.84	3,177	0.001	0.09	Condition 1 > Condition 2; Condition 1 > Condition 4;
Forward saccadic length	*F*_1_	189.06	3,117	<0.001	0.83	Condition 2 > Condition 1; Condition 2 > Condition 3; Condition 2 > Condition 4;
	*F*_2_	258.55	3,177	<0.001	0.81	Condition 3 > Condition 1, Condition 3 > Condition 4; Condition 4 > Condition 1;

*The unit of mean fixation time is milliseconds; the unit of mean saccade length is character; the unit of total time is milliseconds; the unit of reading speed is character per second; the unit of forward saccade length is character. F_1_ means the subject analysis, F_2_ means the item analysis. Condition 1 means the Unspaced format, Condition 2 means the inter-character spaced format, Condition 3 means the inter-word spaced format, Condition 4 means the non-word spaced format.*

The results of time indicators including mean fixation time, number of fixations, total time and reading speed, and space indicators including mean saccadic length and forward saccadic length are presented as follows.

Firstly, in the time metric results, there was a significant word-segmentation effect in the mean fixation duration: *F*_1_(3,117) = 76.6, *p* < 0.001, η^2^ = 0.66; *F*_2_(3,177) = 25.70, *p* < 0.001, η^2^ = 0.30. The post-test results showed no significant difference between the inter-word spaced condition and inter-character spaced condition, *p* > 0.05. There were significant differences between each of the other two conditions, *p_*s*_* < 0.05. The mean fixation duration of the unspaced condition was the longest, *p_*s*_* < 0.001, which implies that the lack of word spaces caused interference in Chinese reading. It is surprising that the facilitation effect of inter-word spaces did not appear in the total time and reading speed. The main effect of word segmentation was significant on total time, *F*_1_(3,117) = 18.01, *p* < 0.001, η^2^ = 0.42; *F*_2_(3,177) = 6.05, *p* = 0.001, η^2^ = 0.09. The *post hoc* test showed that there was not a significant difference between the total time in the unspaced format and the inter-word spaced format, *p_*s*_* > 0.05. This result was consistent with the assumption that the facilitation effect of inter-word spaces changed smaller. Furthermore, there was not a significant difference in the total reading time between the inter-character spaced format and the non-word spaced format, *p_*s*_* > 0.05. This may be the interference of inter-characters offset that of non-word spaces. The difference between each of the other conditions was significant, *p_*s*_* < 0.01. Combined with reading speed, the main effect of word segmentation was significant on reading speed, *F*_1_(3,117) = 20.92, *p* < 0.001, η^2^ = 0.35; *F*_2_(3,177) = 5.84, *p* = 0.001, η^2^ = 0.09. The post-test results showed that the reading speed in the inter-word spaced format was the fastest, but was not significantly different to that in the unspaced format, *p_*s*_* > 0.05. The reading speed in the inter-character spaced format was not significantly different to that in the non-word spaced format, *p_*s*_* > 0.05. However, before the reading training, the reading speed in the inter-word spaced format was significantly faster than that in the unspaced format in Experiment 1, which meant the facilitation of inter-word spaces changed smaller in the Experiment 2.

Secondly, in the results on spacing indicators, we found a significant word-segmentation effect in the number of fixations: *F*_1_(3,117) = 28.31, *p* < 0.001, η^2^ = 0.42; *F*_2_ (3,177) = 21.96, *p* < 0.001, η^2^ = 0.27. The *post hoc* test showed that there was significant difference between each condition, *p_*s*_* < 0.05. Except the comparison between the inter-character word format and the non-word format, which was not significant, *p* = 0.21. The highest number of fixations was found in the inter-character spaced format, *p_*s*_* < 0.05. This may be because the inter-character spaces caused reading interference in Chinese texts. Furthermore, there was a significant word-segmentation effect on the mean saccadic length: *F*_1_(3,117) = 209.98,*p* < 0.001, η^2^ = 0.84; *F*_2_(3,177) = 221.88, *p* < 0.001, η^2^ = 0.79. The *post hoc* test results showed the difference between inter-word spaced format and the non-word spaced format was not significantly, *p* > 0.05. There was a significant difference between each of the other conditions, *p_*s*_* < 0.01. The mean saccadic length in the non-word spaced format was longer than that in the unspaced condition, *p_*s*_* < 0.001. This was because the language information per unit space was the largest under the unspaced format. Furthermore, the main effect of word segmentation on forward saccadic length was significant, *F*_1_ (3,117) = 189.06, *p* < 0.001, η^2^ = 0.83; *F*_2_ (3,177) = 258.55, *p* < 0.001, η^2^ = 0.81. The results in *post hoc* test showed that the difference between each other conditions was significant, *p_*s*_* < 0.001. In sum, the results in the experiment 2 showed that the facilitation of word segmentation changed smaller, which supported research hypothesis.

### Discussion

The purpose of Experiment 2 was to determine whether the facilitation effect of inter-word spaces would change after reading training to improve participants’ familiarity with the format. Combined with the results in the Experiment 1, whether there was a trade-off between format familiarity and the facilitation effect of inter-word spaces was verified.

The results found that the difference between the inter-word spaced format and unspaced format was not significant on the total fixation duration and reading speed, which meant that the facilitation effect of inter-word spaces as word-segmentation clues changed smaller or disappeared. This is consistent with previous studies where no facilitation of inter-word spaces was found for Chinese native readers (Bai et al., 2008, 2012a; Shen et al., 2010; Yan et al., 2012). The common feature of these studies is that the participants were readers with abundant Chinese reading experience, which meant high level of format familiarity in the default Chinese text. Therefore, inter-word spaces did not facilitate the process of Chinese reading when readers had rich experience, that is in the familiar format. In Experiment 2, because reverse-order reading training increased participants’ relevant reading experience, the unfamiliarity of the reverse-order format changed to format familiarity. In a familiar format, the inter-word spaces as word-segmentation clues did not facilitate Chinese reading. The results supported the research hypothesis in Experiment 2: after increasing participants’ familiarity with the format, the facilitation effect of inter-word spaces in Chinese reading changed smaller. There is the other possible explanation, which is “floor effect,” the subjects have reached the best reading performance after reading training in the experiment 2, the inter-word spaces could not facilitate the best reading performance. The “floor effect” may be explain the findings in experiment 2, which could not explain why there is facilitation of inter-word spaces on unfamiliar format in the experiment 1 for Chinese native readers, who also have the best reading ability and best reading performance. The Chinese reading ability would not disappear for just reversing the reading direction. Of course, the “floor effect” should be investigated in the further research via reading training of inter-word spaces for the other subjects.

The combined results of Experiment 1 and Experiment 2 show that there was indeed a trade-off between format unfamiliarity and the facilitation effect of inter-word spaces in Chinese reading, which verified the research assumption (Bai et al., 2008).

## Supplementary Analysis

The facilitation effect of inter-word spaces in a familiar format was examined in Experiment 1 and that in an unfamiliar format was examined in Experiment 2. Combining Experiment1 and Experiment 2, supplementary analysis was conducted to investigate the role of word segmentation and format familiarity in Chinese reading. A repeated-analysis measurement was conducted: 4 (word segmentation: unspaced condition, inter-character spaced condition, inter-word spaced condition, non-word spaced condition) ^∗^ 2 (format familiarity: unfamiliar format and familiar format). The analysis are shown in Table 6.

**TABLE 6:** F values, *p* values, η_*P*_^2^ values of word segmentation and format familiarity and that of Interaction for each index.

Index	Word segmentation			Format familiarity			Interaction		
		*F*	η_*P*_^2^		*F*	η_*P*_^2^		*F*	η_*P*_^2^
Mean fixation duration	*F*_1_	133.58***	0.63	*F*_1_	0.02		*F*_1_	1.88	
	*F*_2_	94.27***	0.62	*F*_2_	2.90		*F*_2_	0.82	
Mean saccadic length	*F*_1_	382.85***	0.83	*F*_1_	22.40***	0.22	*F*_1_	9.37***	0.11
	*F*_2_	300.42***	0.84	*F*_2_	1590.65***	0.96	*F*_2_	8.91***	0.13
Number of fixations	*F*_1_	29.20***	0.27	*F*_1_	57.82***	0.43	*F*_1_	3.06*	0.04
	*F*_2_	12.96***	0.18	*F*_2_	762.48***	0.93	*F*_2_	0.985	
Total fixation duration	*F*_1_	20.01***	0.21	*F*_1_	18.42***	0.19	*F*_1_	4.17**	0.05
	*F*_2_	6.98***	0.11	*F*_2_	789.81***	0.93	*F*_2_	0.77	
Reading speed	*F*_1_	28.17***	0.27	*F*_1_	10.55***	0.12	*F*_1_	4.22**	0.051
	*F*_2_	6.61***	0.10	*F*_2_	657.47***	0.91	*F*_2_	657.47***	0.91
Forward saccadic length	*F*_1_	189.06***	0.83	*F*_1_	294.39***	0.79	*F*_1_	1.53	
	*F*_2_	258.55***	0.81	*F*_2_	278.54***	0.83	*F*_2_	2.61*	0.04

The unit of mean fixation time is milliseconds; the unit of mean saccade length is character; the unit of total time is milliseconds; the unit of reading speed is character per second; the unit of forward saccade length is character. The results in simple-effect were consistent with the results in experiment 1 (unfamiliar format) and experiment 2 (familiar format), which not be included in the Table 6. ***Means P < 0.001, **Means P < 0.01, *Means P < 0.05.

**FIGURE 1:**
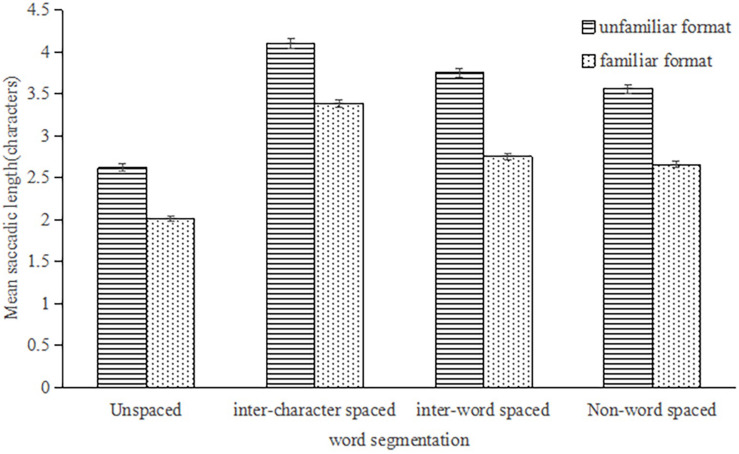
The mean fixation time under the four conditions in the unfamiliar format and familiar format.

The main effect of word segmentation was significant on the mean fixation duration, *F*_1_ (3,234) = 133.58, *p* < 0.001, η_*p*_^2^ = 0.63; *F*_2_ (3,177) = 94.27, *p* < 0.001, η_*p*_^2^ = 0.62; the main effect of format familiarity on the mean fixation duration was not significant, *F*_1_(1,78) = 0.02, *p* > 0.1; *F*_2_ (1,59) = 2.902, *p* = 0.09; the interaction between word segmentation and format familiarity was not significant, *F*_1_ (3,234) = 1.88, *p* > 0.1; *F*_2_(3,177) = 0.824, *p* > 0.1. See Figure 1.

**FIGURE 2:**
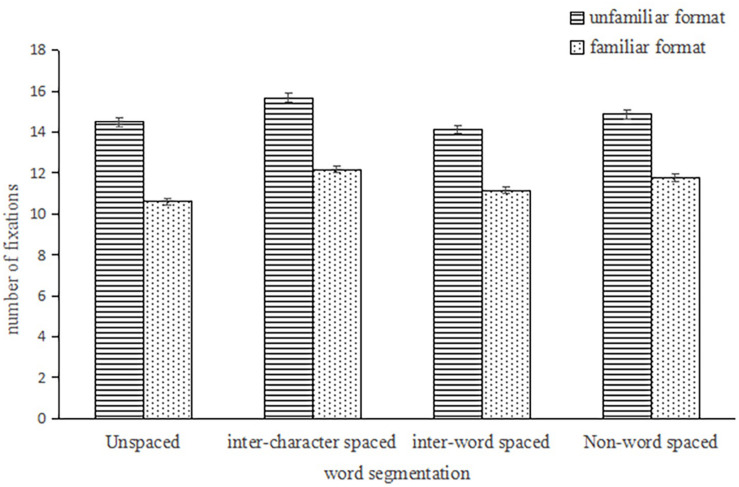
The mean saccadic length under four conditions in the unfamiliar format and familiar format.

The main effect of word segmentation on the mean saccadic length was significant, *F*_1_(3,234) = 382.85, *p* < 0.001, η_*p*_^2^ = 0.83; *F*_2_(3,177) = 300.42, *p* < 0.001, η_*p*_^2^ = 0.84; the main effect of format familiarity was significant, *F*_1_(1,78) = 22.40, *p* < 0.001, η_*p*_^2^ = 0.22; *F*_2_ (1,59) = 1590.65, *p* < 0.001, η_*p*_^2^ = 0.96; the interaction between word segmentation and reading training was significant, *F*_1_(3,234) = 9.37, *p* < 0.001, η_*p*_^2^ = 0.11; *F*_2_ (3,177) = 8.91, *p* < 0.001, η_*p*_^2^ = 0.13. See Figure 2. A simple-effects analysis found that the main effect of word segmentation on the unfamiliar format was significant, *F*_1_ (3,76) = 139.16, *p* < 0.001, η_*p*_^2^ = 0.85; *F*_2_ (3,57) = 155.64, *p* < 0.001, η_*p*_^2^ = 0.89. In the unfamiliar format, there was a significant difference between each condition (*p*_*s*_ < 0.01). On the other hand, there was a significant difference between the four conditions in the familiar format, *F*_1_(3,76) = 81.83, *p* < 0.001, η_*p*_^2^ = 0.76, *F*_2_ (3,57) = 327.16, *p* < 0.001, η_*p*_^2^ = 0.95. There was only one difference in the familiar format, where the difference between inter-word spaced format and non-word spaced format was not significant, *p* = 0.24. This meant the interference of non-word and facilitation of inter-word reduced were decreased at the same time on the familiar format.

**FIGURE 3:**
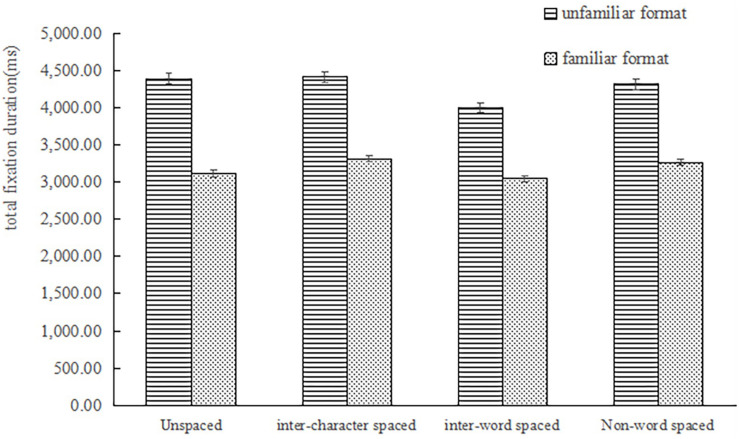
The number of fixations under four conditions in the unfamiliar format and familiar format.

There was a significant word-segmentation effect [*F*_1_(3,234) = 29.198, *p* < 0.001, η_*p*_^2^ = 0.27; *F*_2_ (3,177) = 12.96, *p* < 0.001, η_*p*_^2^ = 0.18] and a significant format-familiarity effect [*F*_1_(1,78) = 57.82, *p* < 0.001, η_*p*_^2^ = 0.43; *F*_2_ (1,59) = 762.48, *p* < 0.001, η_*p*_^2^ = 0.93] on the number of fixations. The interaction was significant on the subjective analysis, *F*_1_ (3,234) = 3.06, *p* < 0.05, η_*p*_^2^ = 0.04; *F*_2_ (3,177) = 0.985, *p* = 0.401. See Figure 3. The simple-effects analysis found that there was a significant word-segmentation effect on the unfamiliar format, *F*_1_(3,76) = 16.25, *p* < 0.001, η_*p*_^2^ = 0.39; *F*_2_(3, 57) = 4.14, *p* = 0.01, η_*p*_^2^ = 0.18. The subject-analysis results showed that the number of fixations in the inter-character spaced condition was larger than the other conditions (*p_*s*_* < 0.01). However, there was no significant difference between the unspaced condition and the inter-word spaced condition (*p* = 1.00), and the difference between the unspaced and the non-word spaced conditions was not significant (*p* = 0.60). There were significant differences between each of other conditions (*p_*s*_* < 0.01). The item analysis results were not totally consistent with those of the subject analysis. Firstly, there was no significance between the unspaced condition and the inter-character spaced condition (*p* = 0.14). Secondly, there was not significant between the inter-character spaced and non-spaced condition (*p* = 1.00). On the other hand, there was a significant word-segmentation effect on the familiar format, *F*_1_(3, 76) = 16.42, *p* < 0.001, η_*p*_^2^ = 0.39; *F*_2_(3, 57) = 16.51, *p* < 0.001. η_*p*_^2^ = 0.47. The subject-analysis results showed that the number of fixations in the inter-character spaced condition was the largest, with no significant difference with that in the non-word spaced condition (*p* = 0.23). At the same time, there was no significant difference between the unspaced condition and the inter-word spaced condition (*p* = 0.12). There were significant differences between each other condition (*p_*s*_* < 0.05). The item analysis results were not totally consistent with those of the subject analysis. Firstly, there was no significant difference between the unspaced condition and the inter-character spaced condition (*p* = 0.23). There were significant differences between each of the other conditions (*p_*s*_* < 0.05).

**FIGURE 4:**
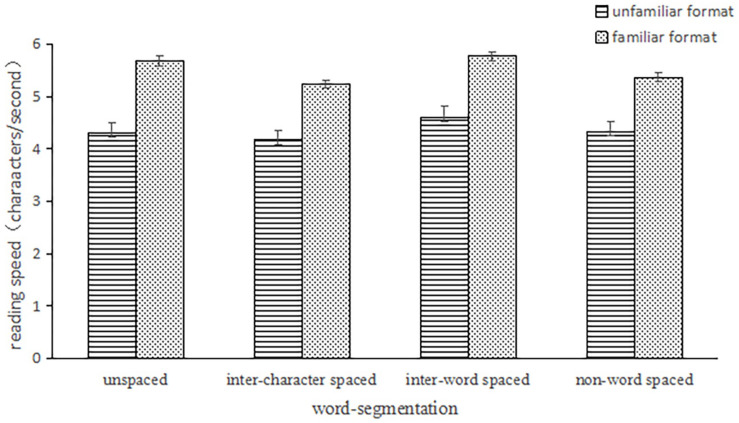
The total fixation time under four conditions on the unfamiliar format and familiar format.

The main effect of word segmentation [*F*_1_(3,231) = 20.01, *p* < 0.001, η_*p*_^2^ = 0.21; *F*_2_(3,177) = 6.98, *p* < 0.001, η_*p*_^2^ = 0.11) and format-familiarity [*F*_1_(1,77) = 18.42, *p* < 0.001, η_*p*_^2^ = 0.19; *F*_2_(1,59) = 789.81, *p* < 0.001, η_*p*_^2^ = 0.93] on the total fixation duration were significant. The interaction was significant in the subject analysis but not in the item analysis, *F*_1_(3,231) = 4.17, *p* < 0.01, η_*p*_^2^ = 0.05; *F*_2_ (3,177) = 0.77, *p* = 0.51. See Figure 4. A simple-effects analysis found the main effect of word segmentation was significant in the unfamiliar format, *F*_1_ (3, 75) = 14.71, *p* < 0.001, η_*p*_^2^ = 0.37. The difference between unspaced format and inter-character spaced format was not significant (*p* = 1.00), and that between unspaced format and non-spaced format was not significant (*p* = 1.00). These difference was significant (*p* = 0.008) or marginally significant (*p* = 0.06) in the experiment 2, which meant the interference of inter-character spaces and non-word spaces was appeared on the familiar format. Interestingly, the facilitation of inter-word spaces changed smaller in the familiar format. The total fixation duration under the inter-word spaced format was the significantly shortest (*p_*s*_* < 0.001). However, the difference between the inter-word spaced format and unspaced format was not significant on the familiar format (*p* = 1.00). The other results under familiar format were consistent with the results under unfamiliar format.

**FIGURE 5:**
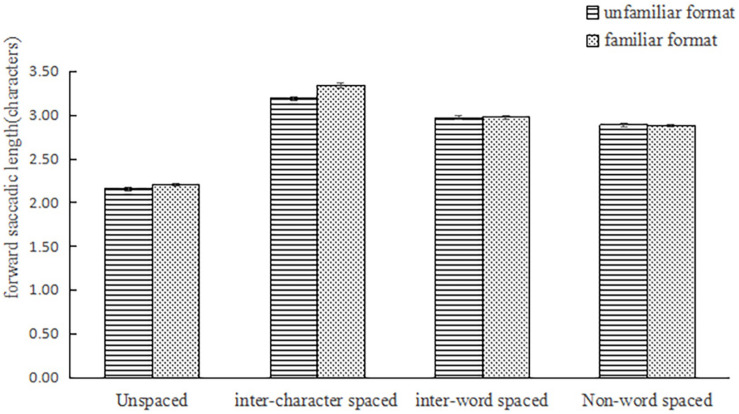
The reading speed under four conditions on the unfamiliar format and familiar format.

The main effect of word segmentation [*F*_1_(3,234) = 28.17, *p* < 0.001, η_*p*_^2^ = 0.27; *F*_2_(3,177) = 6.61, *p* < 0.001, η_*p*_^2^ = 0.10] and format-familiarity [*F*_1_(1,78) = 10.55, *p* < 0.001, η_*p*_^2^ = 0.12; *F*_2_(1,59) = 657.47, *p* < 0.001, η_*p*_^2^ = 0.91] on reading speed were significant. The interaction was significant in the subject analysis but not on the item analysis, *F*_1_(3,234) = 4.22, *p* < 0.01,η_*p*_^2^ = 0.051; *F*_2_(3,177) = 0.68, *p* > 0.1. See Figure 5. Simple-effects analysis found the main effect of word segmentation was significant in the unfamiliar format, *F*_1_(3, 76) = 12.25, *p* < 0.001, η_*p*_^2^ = 0.33; the subject-analysis results found the reading speed in the inter-word spaced condition was significantly faster than the other conditions, *p_*s*_* < 0.001. The difference between unspaced format and inter-word spaced format was no more significant under familiar format (*p* = 1.00), which implied that the facilitation of word segmentation was changed smaller. Interestingly, the difference between the unspaced format and inter-character spaced format was not significant under unfamiliar format, which was significant under familiar format. This implied that the interference of inter-character spaces only appeared under the familiar format. On the other hand, there was significant differences under familiar format, *F*_1_(3,76) = 19.46, *p* < 0.001, η_*p*_^2^ = 0.43. The subject-analysis results showed that the difference in the unspaced and inter-word spaced condition was not significant, which means that the facilitation of word segmentation changed smaller under familiar format.

**FIGURE 6:**
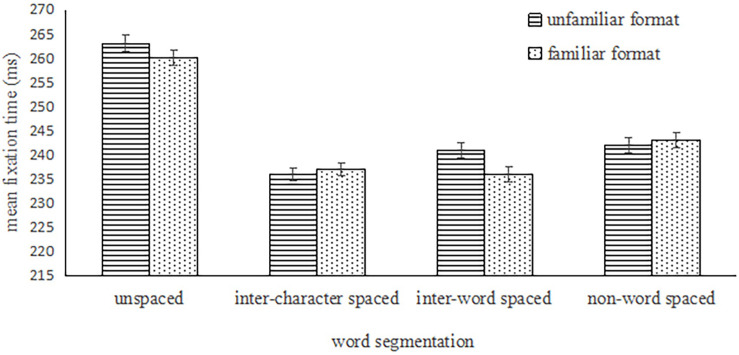
The forward saccadic length under four conditions on the unfamiliar format and familiar format.

The main effect of word-segmentation conditions [*F*_1_(3,234) = 294.39, *p* < 0.001, η_*p*_^2^ = 0.79; *F*_2_(3,177) = 278.54, *p* < 0.001, η_*p*_^2^ = 0.83] on forward saccadic length was significant; however, it was not significant on format-familiarity [*F*_1_ (1,78) = 0.000, *p* > 0.1; *F*_2_ (1,59) = 11.27, *p* = 0.001, η_*p*_^2^ = 0.16]. The interaction was not significant in the subject analysis, but was marginally significant in the item analysis, *F*_1_ (3,234) = 1.53, *p* > 0.1; *F*_2_ (3,177) = 2.61, *p* = 0.05, η_*p*_^2^ = 0.04. See Figure 6. Simple-effects analysis found that the main effect of word segmentation was significant in the unfamiliar format, *F*_2_(3, 116) = 279.94, *p* < 0.001. The subject-analysis results found that the difference was significant in the other conditions (*p_*s*_* < 0.001) except for the inter-word spaced and non-word spaced conditions (*p* > 0.05).

## General Discussion

This study aimed at investigating whether there was a trade-off between format familiarity and word-segmentation facilitation in Chinese reading. The reading training was manipulated to control the format familiarity, and EyeLink 1000 (SR Research, Canada) was used to record the eye tracking of adult participants in four word-segmentation conditions. The results showed there was a trade-off between format unfamiliarity and word-segmentation facilitation in Chinese reading.

Based on this, the primary task for readers is to segment words in unspaced texts in Chinese reading. However, a consensus has not been reached over whether inserting spaces between words facilitates Chinese reading. Bai et al. (2008) did not find that inter-word spaces facilitated the reading of native Chinese undergraduates, which was explained on the basis of a trade-off between inter-word spaces and format familiarity. The facilitating effect was offset by the format unfamiliarity of inter-word spaces. However, in the unspaced condition, where there was neither facilitation of word segmentation or format unfamiliarity, and there was no significant difference in reading performance. Therefore, the format familiarity was controlled in Experiment 1. For readers, the unspaced format and inter-word spaced format in the Chinese reverse texts read from right to left were unfamiliar formats. The results showed that inter-word spaces facilitated reading in the unfamiliar format, where the reading speed in the inter-word spaced condition was faster than that in the unspaced condition. The results supported the prior assumption and were consistent with the research hypothesis. Based on this finding, Experiment 2 was designed to investigate whether the facilitation effect would change, that was disappear or reduce. We improved participants’ familiarity with the unspaced text format which was just like normal Chinese texts via reading reverse Chinese texts for 30 min per day for 10 days. In Experiment 2, the eye tracking of new participants reading the Chinese sentences was recorded; the reason for changing the participants was to retain the same Chinese sentences in the eye-movement experiment. Surprisingly, the results found that there was no significant difference between the reading speed and total fixation time in the unspaced and inter-word spaced conditions. This meant that the facilitation effect of inter-word spaces as word-segmentation indicators changed smaller. The combined results in Experiment 1 and Experiment 2 showed a trade-off between format familiarity and word-segmentation facilitation in Chinese reading.

The default format of the reading and writing system in contemporary Chinese language is from left to right, which is regarded as a familiar format by native Chinese readers. There is a trade-off between format familiarity and the facilitation effect of inter-word spaces in Chinese reading; native Chinese readers have a high level of format familiarity, and so the facilitation of word segmentation did not appear as in prior studies (Bai et al., 2008; Shen et al., 2010; Li et al., 2011b; Yan et al., 2012). The common feature of these previous studies was that the participants had sufficient reading experience to offset the word-segmentation facilitation. This provides a reasonable explanation for this finding. On the other hand, the previous studies which found that inter-word spaces facilitated Chinese reading had a common feature; that is, the participants who were foreign undergraduates or Chinese children with dyslexia had insufficient Chinese reading experience. Lack of Chinese reading experience caused format unfamiliarity, which could not offset the inter-word facilitation. Therefore, there was word-segmentation facilitation of the Chinese reading process in these studies (Bai et al., 2009, 2011; Wang et al., 2010; Blythe et al., 2012; Shen et al., 2012; Gu et al., 2017). The inconsistency of word-segmentation facilitation comes from having sufficient Chinese reading experience. The reverse Chinese texts presented single characters from right to left, and then the readers needed to read from right to left in an unfamiliar format. Therefore, this study used the reading training of reverse Chinese texts in an unfamiliar format, which improved the relevant reading experience for readers. In Experiment 2, the results showed that the facilitation effect of inter-word spaces found in Experiment 1 changed smaller via improving the participants’ familiarity with the format of reverse Chinese texts. In sum, the results supported the research hypothesis.

In addition, the trade-off can also explain the assumption in the previous studies, which proposed that inter-word spaces could play a certain role in promoting Chinese reading of difficult or ambiguous texts (Hsu and Huang, 2000; Inhoff et al., 2000; Inhoff and Radach, 2002; Li et al., 2011a). Hsu and Huang (2000) found that inter-word spaces as word-segmentation indicators help readers to achieve faster word recognition and reading comprehension compared with unspaced texts. Compared with the default format, the readers have to pay a higher reading cost to process the reverse Chinese texts, where the reading difficulty was higher. This means that the inter-word spaces could facilitate Chinese reading in difficult texts.

The combined results in Experiment 1 and Experiment 2 showed that a trade-off between format unfamiliarity and word-segmentation facilitation of Chinese reading did indeed exist. For unspaced Chinese texts, word segmentation did not depend on the low-level visual clues. The mechanisms behind the trade-off were the low-level visual factor (format familiarity) and the high-level cognition factor (reading experience). The mechanism can be explained by the holistic hypothesis of the computational model on the word-segmentation mechanism in Chinese reading (Li et al., 2009). Two assumptions were included in the model: feed-forward assumption and holistic hypothesis. The first assumption supported the process whereby the character recognition system obtains visual information from characters and then transfers it to the word-segmentation stage, and finally integrates it into the word-recognition stage. There is only feed-forward from bottom to top and no feedback from top to bottom in word processing. However, the holistic hypothesis supports the notion that the visual information system, character recognition system and word-recognition system affect the word-segmentation stage and word-recognition stage interactively. Previous studies supported the holistic assumption being suitable for Chinese reading, which is consistent with the results in this study (Li et al., 2009, 2011a; Ma, 2017; Ma and Zhuang, 2018). Based on the holistic hypothesis, the inter-word spaces as low-level visual information affect reading comprehension from bottom to top, while the reading experience as a high-level cognition factor affects reading comprehension from top to bottom. Therefore, the reading experience as a high-level cognitive factor behind format familiarity and inter-word spaces as a low-level visual factor would both affect the Chinese reading, with a trade-off between them. The assumption explained that there was no facilitation effect of inter-word spaces in the previous studies, where the participants were native adults with rich Chinese reading experiences (Bai et al., 2008; Shen et al., 2010; Yan et al., 2012; Liu and Li, 2014). The richness of Chinese reading experiences affected the facilitation of inter-word spaces as the low-level visual clues in word recognition and reading comprehension. However, previous studies of foreign students who learned Chinese as a second language found that the inter-word spaces facilitated reading speed and prompted word recognition as well as reading comprehension. This is because Chinese learning beginners had insufficient Chinese reading experience, and the inter-word spaces could help them to segment word from Chinese texts, so as to acquire faster reading comprehension and word recognition (Wang et al., 2010; Shen et al., 2012; Gu et al., 2017; Zhou et al., 2020).

In the current study, the reading training just contained unspaced reverse texts to improve the format familiarity. Further research could focus on improving the format familiarity of inter-word spaced text via reading training in Chinese reverse texts where spaces have been inserted between words. Whether the facilitation effect of inter-word spaces would appear after improving the format familiarity of inter-word spaced text needs to be further verified. In addition, future studies could manipulate the inter-word spaces, word frequency and format familiarity to explore the relationship between them and examine the mechanism between word segmentation and word recognition. Moreover, The expect direction is the trade-off during learning a new language in the future, especially for non-native readers, when they start to learn Chinese which is not familiar orthography and semantic information, the format familiarity may have more influence on the facilitation of the inter-word spaces. For example, Uyghur whose mother tongue is presented from right to left start to learn Chinese, however, they are unfamiliar with the Chinese format from left to right. The reading performance of Uyghur and that of Chinese could be compared to expect new surprising findings.

## Conclusion

To summarize, the results indicate that inter-word spaces as low-level visual clues facilitate Chinese reading in an unfamiliar format. After improving participants’ familiarity with the format, the facilitation effect of inter-word spaces changed smaller. There is a trade-off between format familiarity and facilitation of inter-word spaces, which supported the assumption made in previous studies.

## Data Availability Statement

The original contributions presented in the study are included in the article/Supplementary Material, further inquiries can be directed to the corresponding author/s.

## Ethics Statement

Based on the Declaration of Helsinki (BMJ 1991; 302: 1194), Tianjin Normal University’s Medical Ethical Committee approved the experiment. The patients/participants provided their written informed consent to participate in this study.

## Author Contributions

XB provided the research idea and the research construction. MC and YW designed the experiments and wrote the manuscript. MC conducted the data analysis. BZ participated the eye-movement experiment. XL contributed to writing the manuscript. All authors contributed to the article and approved the submitted version.

## Conflict of Interest

The authors declare that the research was conducted in the absence of any commercial or financial relationships that could be construed as a potential conflict of interest.
