# Corrigendum: The Trade-Off Between Format Familiarity and Word-Segmentation Facilitation in Chinese Reading

**DOI:** 10.3389/fpsyg.2021.665606

**Published:** 2021-03-25

**Authors:** Mingjing Chen, Yongsheng Wang, Bingjie Zhao, Xin Li, Xuejun Bai

**Affiliations:** Key Research Base of Humanities and Social Sciences, Institute of Psychology and Behavior, Tianjin Normal University, Tianjin, China

**Keywords:** trade-off, format familiarity, word segmentation, Chinese reading, eye movements

In the original article, we neglected to include the following funding information:

‘Ministry of Education, Humanities and Social Sciences Research Youth Project, China, 18YJC190023.’

The authors apologize for this error and state that this does not change the scientific conclusions of the article in any way. The original article has been updated.

