# Supplementary material for: The Trade-Off Between Format Familiarity and Word-Segmentation Facilitation in Chinese Reading
Source: Front Psychol. 2021 Jan 28;12:602931. doi: 10.3389/fpsyg.2021.602931 (PMC7876460; doi:10.3389/fpsyg.2021.602931)
Supplement: Supplementary file 1 [file Data_Sheet_1.doc]

Appendix：The example of essay used in the experiment 2

手到剪心小 1

怎可己自到剪，下放快，哎宗祖小：了坏吓奶奶的他顾照把可，刀剪起拿次一第子孩

。刀剪了下放地乖乖子孩？了得么

准正，刀剪和笔画、纸来找地奋兴子孩，后之家回，工手了教师老，了园儿幼上子孩

要你，险危太，刀剪么什玩子孩小：刀剪的中手子孩了过夺把一，了现出妈妈，时纸剪备

一。来起多课工手，了学上子孩。妈妈了给交刀剪将地悻悻子孩。剪你帮妈妈，么什做

见看爸爸，刀剪起拿刚子孩。妈妈给送，品艺工手个一做手动，业作庭家了置布师老，次

。刀剪了下放，手的己自看看又，刀剪的晃晃亮看看子孩。手到剪心小，子孩嘱叮，了

妈”？儿哪在刀剪“妈妈问。了剪头线把刀剪用备准，头线个一有上服衣，现发子孩

是咬，头线咬地力吃头低，下一了豫犹子孩？了以可就不掉咬牙用，么什干刀剪要，说妈

剪起拿刚，下一剪修角个四将刀剪用想，皮书好包子孩 。子样成不得咬角衣将也，了掉咬

指手将心小，刀剪用要不万千，说妈妈，了长甲指的子孩。手到剪心小，呼惊就妈妈，刀

，撕？呢它开打么怎，是可，食零的致精装包袋一着拿，中手的子孩。钳甲指用，了掉剪

主了有于终他，后最。了来出流快都泪眼得急子孩……掉不拽，拽；开不咬，咬；动不撕

……了开剪就剪一轻轻，刀剪起拿妈妈。开剪他帮妈妈让，刀剪和袋食零着拿，意

近有然竟班全，工手做们子孩教她，次一。事件一起讲我跟，友朋的师老当位一的我

用不也，看难分十，扭扭歪歪得撕纸将，撕手用愿宁们他，刀剪用敢不或会不子孩的数半

没，了大么这能可么怎。过用没，答回子孩？吗刀剪过用没里家在道难，们他问她。刀剪

敢不但用会个一。险危说，刀剪用让不妈妈爸爸，答回子孩。们他问地异诧她？刀剪过用

这都次每，手到剪心小，说我对会就人大，刀剪起拿一我要只次每，说充补子孩的刀剪用

好友朋？办么怎候时的刀剪用要需们你果如那。了刀剪用不也再就性索我是于，烦很，样

。呗人大找？单简不还这，了笑们生学。生学问地奇

安意注何如他知告并，用么怎他教地心耐是？的理处么怎是你，候时的刀剪起拿子孩

起拿子孩在次每会不会你？忙帮去己自，刀剪用使子孩让绝拒而，险危免避了为是还，全

的意善句一是来本这，手到剪心小？”手到剪心小“他嘱叮再一地心放不都，候时的刀剪

不，查调小个一过做人有。力压和担负的形无种一了成就，说来子孩对，了多说但，醒提

都，候时的刀剪起拿次每們他在，子孩的刀剪用使愿不或，刀剪用使敢不或，刀剪用使会

孩的有；了刀剪用不性索，烦其胜不子孩的有，是于。咛叮句这的辈长他其或母父到听会

刀剪碰敢不也再是更此从，手了到剪的真心小不，子孩的有还；了刀剪用不也，了怕害子

大，巴泥玩子孩。跤摔心小，子孩咛叮人大，跑奔子孩。上刀剪在出仅仅不然当题问。了

，碗洗子孩。来下摔心小，子孩训教人大，高攀或树爬子孩。服衣脏弄心小，子孩斥呵人

。了走拐人被心小，子孩唬吓人大，门出人个一子孩。碟碗了碎打心小，说地心放不人大

不会人大，西东么什个一装组图试子孩。去回不装心小，子孩骂人大，具玩的他散拆子孩

……么什腾折瞎糟八七乱，屑

是你实其。任责的己自尽在你为以你，好子孩为是都这为以你，子孩护呵是你为以你

把这用他了止阻你但，误错和险危了离远他让你来起看，”刀剪“把那的上手子孩了走拿

最新最出剪修么怎还他，力能造创和手动的他了缚束你，手的巧灵该本他了住捆你，刀剪

的他有还，刀剪把一是单不的起拿他。候时的刀剪起拿子孩在，手放？呢活生和案图的美

。来未

？刀剪用敢不学同少多概大上班现发友朋师老位一的“我“：一题问

学同部全:A

学同些一:B

学同数半近:C

？刀剪用敢不学同数半近么什为：二题问

刀剪用欢喜不就身本们他:A

让不长家:B

撕惯习更们他:C

。况情的样这过到遇曾都，学同的刀剪用使愿不、敢不、会不，查调过做人有：三题问

过害伤刀剪被:A

嘱叮的辈长或母父到听会都，时刀剪起拿次每:B

人他过害伤刀剪用:C

来未的他有还，刀剪把一是单不的起拿他。候时的刀剪起拿子孩在“说么什为：四题问

力造创的子孩碍阻会能可有，误错和险危离远子孩让的度过为因:A

康健体身的来未他响影会这，己自害伤刀剪用子孩:B

能可的来未拓开有具就，气勇的刀剪用使有具子孩:C

______？吗章文篇这读阅次一第是你：五题问

是不.B 是.A

_______? 吗懂读易容章文篇这为认你：六题问

易容不常非.D 易容不.C 易容.B 易容常非.A

________？吗容内的章文篇这欢喜你：七题问

欢喜不常非.D 欢喜不.C 欢喜.B 欢喜常非.A
